# The Chemistry and Pharmacology of the Alkaloid Barettin and Its Analogues from the Marine Sponge *Geodia barretti*: Progress and Perspectives

**DOI:** 10.3390/md24030110

**Published:** 2026-03-13

**Authors:** Christian Bailly

**Affiliations:** 1 CHU de Lille, U1366 Inserm, UMR9020 CNRS, University of Lille, 59006 Lille, France; christian.bailly@univ-lille.fr; 2 Institute of Pharmaceutical Chemistry Albert Lespagnol (ICPAL), Faculty of Pharmacy, University of Lille, 59006 Lille, France; 3 OncoWitan, 59290 Lille, France

**Keywords:** *Geodia barretti*, barettin, marine sponge, diketopiperazine, antifouling agents

## Abstract

The cold-water siliceous sponge *Geodia barretti*, largely present in the North Atlantic Ocean, notably around Scandinavian costs, plays important roles in carbon and silicon cycling in the deep-sea. The demosponge provides a reservoir for numerous microorganisms. Bioactive natural products have been isolated from this sponge, in particular the indole alkaloid barettin discovered forty years ago. Barettin and analogues, notably 8,9-dihydrobarettin, 8,9-dihydro-8-hydroxybarrettin, bromobenzisoxalone barettin, and geobarrettins A-B, contribute to the maintenance of the sponge stability and security (anti-fouling) and the regulation of its microbial environment. The four indole alkaloids 6-bromo-8-hydroxyconicamin, 6-bromoconicamin, and geobarrettin C-D are also implicated in the defense of the sponge against physical and biochemical aggressions. Altogether, these ten natural products are essential to the sponge life. The present review presents a survey of the chemistry and biology associated with *Geodia barretti*. The pharmacological properties of (dihydro)barettin, notably their antioxidant and anti-inflammatory properties, are discussed, as well as the synthetic processes set up to produce these diketopiperazine derivatives. Their molecular targets and mechanism of action are also discussed. The review takes the sponge *G. barretti* from the depths of knowledge and brings barettin and analogues to the surface, with the hope of guiding future research in this field.

## 1. Introduction

Marine organisms provide a large reservoir of bioactive natural products to combat human diseases. Numerous substances of pharmacological interest have been isolated from marine species, including macro- (e.g., molluscs, sponges, coral, mangrove plants, etc) and micro-organisms such as marine algae, bacteria, fungi, and phytoplankton [1,2,3,4]. Novel products of medicinal interest are continuously identified and pharmacologically characterized. The marine natural product research field is very dynamic, with over 1200 new products identified in both 2023 and in 2024, with relevant biological activities [5,6]. The marine environment offers unique molecular entities which can be exploited to combat a large range of diseases, including neurodegenerative, cardiovascular, pulmonary, and musculoskeletal metabolic diseases; cancers; and infectious pathologies [7]. In addition, marine products are of significant interest in the fields of nutrition, ecology, energy, agri/aqua-culture, and other domains [8]. Aquatic ecosystems are essential to life but they are continuously stressed by anthropogenic activities and increasingly altered and threatened by diverse pollutants [9].

Among marine organisms and marine invertebrates in particular, the phylum of sponges (Porifera) has been well studied as a source of bioactive molecules due to the large species diversity and the relative ease of collecting them compared to mobile organisms. An enormous diversity of bioactive products has been found in those sessile organisms, and their associated microorganisms, notably terpenes, alkaloids and peptides [10,11]. Potent anticancer, antibacterial and anti-inflammatory products have been isolated from marine sponges in recent years [12,13,14]. In particular, interesting bioactive products have been found in marine sponges of the genus *Geodia* which includes a large number of species (>160) only living in deep waters [15]. The leading species in this group is *Geodia barretti* (Geodiidae), from which diverse bioactive natural products have been isolated, in particular the 2,5-diketopiperazine barettin, endowed with marked anti-inflammatory properties. In recent years, the chemistry of this marine alkaloid has been developed to facilitate access to the natural product and derivatives. In parallel, the biology and pharmacology of barettin have been investigated and promising results have been reported with a few derivatives. It is timely to present an overview of medicinal properties of barettin to guide future research.

## 2. Review Methodology

An exhaustive analysis of the scientific literature pertaining to the sponge *Geodia barretti* and derived natural products structurally related to barettin has been performed. All available publications (essentially in English) and reports (including PhD theses) available from scientific data banks (PubMed, Scopus, Web of Science, and Google Scholar) have been consulted. Keywords were used to identify relevant publications, notably *Geodia* and barettin (as well as barrettin, the name of the natural product often being misnamed in publications). A PRISMA-type methodology was followed (>250 initial records, 140 studies included). The goal of the analysis was to perform a comprehensive analysis of (i) the biology and chemistry of *G. barretti*, and (ii) the chemistry and pharmacology of barettin and related products, natural or synthetic. All products of bio- and pharmacological interest isolated from the sponge are discussed, with a focus on the barettin family, with the goal to raise ideas for future research in this field.

## 3. From *Geodia barretti* to Barettin and Related Natural Products

**Figure 1:**
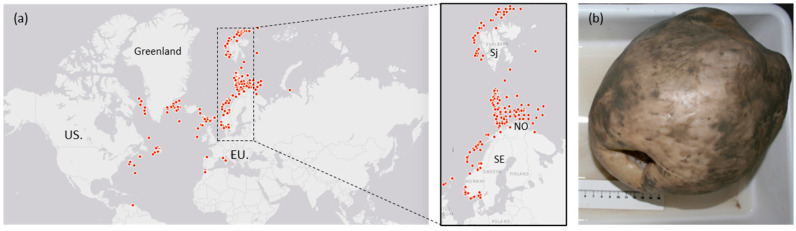
(**a**) World distribution of *Geodia barretti* Bowerbank, 1858. The sponge is mainly located in the North Atlantic Ocean, notably around the coasts of Norway (including around the Svalbard (Sj) Artic archipelago) and Sweden. Map from Ocean Biodiversity Information System (OBIS) (https://mapper.obis.org/?taxonid=134023# (accessed on 19 February 2026)). (**b**) Photo of the sponge, from the Word Register of Marine Species (WoRM) (https://www.marinespecies.org/aphia.php?p=taxdetails&id=134023 (accessed on 19 February 2026)).

The demosponge species *Geodia barretti* Bowerbank, 1858 (hereafter *G. barretti*) is well distributed in the North Atlantic Ocean (Figure 1a). Its vernacular name is Barrett’s horny sponge, in honor of the English naturalist Lucas Barrett (1837–1862) who discovered the sponge (together with Robert MacAndrew) in Norway (circa 1858). This sponge can be found in Scandinavian waters, where massive colonies (80 cm in diameter) have been observed [16,17]. It is present also on the North American continental shelf, notably on underwater plateaus south-east of the island of Newfoundland [18]. The sponge presents a round excretory structure (osculum) with a hole on top through which the current of water exits (Figure 1b). It grows well in deep-sea environments, at a depth of about 1200–1400 m, where it contributes to nitrogen turnover and nitrogen mineralization [19,20]. *G. barretti* species are often organized in clusters in which these dioecious and oviparous sponges proliferate. Colonies are represented by dense assemblages of organisms which have an annual reproductive cycle with one or two periods of gamete release per year [21].

A rich microbial community is associated with the sponge, notably ammonia-oxidizing bacteria and archaea [22,23]. The *G. barretti* microbiome varies with the metabolic status of the sponge. A marked change in the microbial abundance has been observed in brown/black discolored, diseased sponges with a higher amount of Bacteroidetes, Firmicutes and Deltaproteobacteria in diseased individuals compared to healthy sponges [24,25]. This sponge exhibits high thermal tolerance, but it is sensitive to acute thermal stress events and to contaminants such as barite and bentonite present in drilling muds [26,27,28]. A recent study revealed that exposure to suspended particles of seafloor massive sulphide (SMS) induced necrosis of the sponge with an accumulation of metals (Fe,Cu) in the sponge’s mesohyl [29].

**Figure 2:**
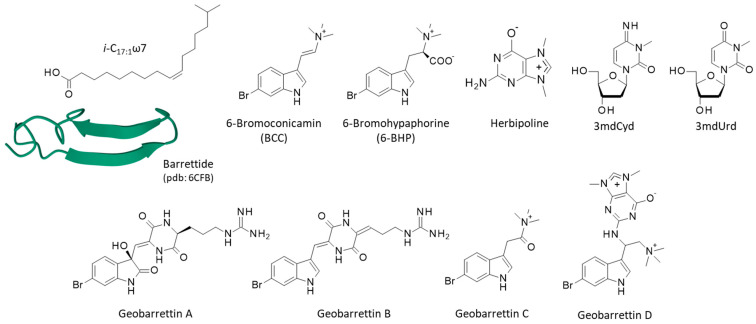
Structures of metabolites isolated from *G. barretti*. (3mdCyd = 3-methyl-2′-deoxycytidine; 3mdUrd = 3-methyl-2′-deoxyuridine). The iso-branched monounsaturated fatty acid *i*-C_17:1_ω7 (MUFA omega-7) is a representative member of the series of fatty acids found in the sponge. Twenty-six other fatty acids (notably 8- and 9-Me-C_16:0_, C_16:1_ω7, *i*-C_15:0_ and C_26:2_Δ^9,19^ (ω7)) have been identified from *G. barretti* [31]. The NMR-based model (in green) of the 31-amino acid, disulfide-containing peptide barrettide A was obtained from the protein data bank (pdb) under the code 6CFB (https://www.rcsb.org/structure/6CFB (accessed on 19 February 2026)) [33].

The whole genome of *G. barretti* has been sequenced and annotated. It includes 31,884 protein-coding genes and a mitochondrial genome of 17,996 bp [30]. The sponge can produce a large diversity of secondary metabolites including (i) long-chain (C24–C28) linear and branched unsaturated fatty acids [31], (ii) large peptides, such as barrettides A-G endowed with antifouling activities [32,33], (iii) N-methylated nucleosides (3-methyl-2′-deoxycytidine and 3-methyl-2′-deoxyuridine) [34,35] and (iv) small molecules in the form of cyclic dipeptides and indole alkaloids, such as 6-bromoconicamin and 6-bromohypaphorine (6-BHP) [36] (Figure 2). This latter product 6-BHP is an amino acid-type indole alkaloid also found in other marine sponges (e.g., *Aplysina* species, *Pachymatisma johnstoni*) [37,38] and in the red sea cucumber *Apostichopus japonicus* [39]. This compound functions as an agonist of human α7 nicotinic acetylcholine receptors (nAChR), which is a ligand-gated ion channel widely expressed in the central nervous system and in the heart in humans [40]. Synthetic analogues of 6-BHP with a selective and reinforced agonist activity toward α7 nAChR have been designed. They are investigated for the treatment of myocardial infarction and inflammatory diseases [41,42]. 6-Bromoconicamin (BCC) and the derivative 8-hydroxy-6-bromoconicamin have been identified from the sponge [43]. BCC is a modest inhibitor of acetylcholinesterase but a potent inhibitor of butyrylcholinesterase (IC_50_ = 230 and 14 µM, respectively), equally efficient as the reference cholinesterase inhibitor galanthamine [44].

**Figure 3:**
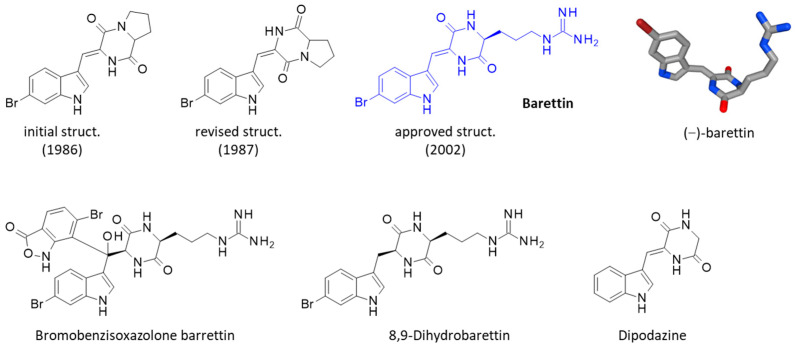
Structures of barettin and derivatives. Structures of (−)-barettin (C_17_H_19_BrN_6_O_2_) and 8,9-dihydrobarettin (cyclo[(6-bromotryptophan)arginine], C_17_H_21_BrN_6_O_2_) are represented.

The brominated alkaloids barettin and 8,9-dihydrobarettin are the two key products found in *G. barretti* (Figure 3). They are brominated diketopiperazine-like cyclic dipeptides endowed with antifouling activities and serving as a chemical defense against mollusk grazers [45,46]. Barettin was first isolated from the sponge in 1986 [47]. Initially, its structure (C_15_H_14_N_3_O_2_Br) was assigned on the basis of analytical (NMR) data and a comparison with cyclo(L-Pro-L-Trp), which is also known as brevianamide F [47]. A second structure was proposed one year later [48]. The diketopiperazine structure initially assigned to barettin has been reconsidered in 2002. A new structure corresponding to the condensation product of 6-bromotryptophan and arginine has been proposed and validated (C_17_H_19_N_6_O_2_Br). Barrettin presents a guanidine side chain attached to the piperazinedione unit (Figure 3) [49]. 8,9-Dihydrobarettin is the brominated analogue of cyclo(L-Arg-L-Trp) (cRW) biosynthesized by certain fungi, notably *Aspergillus versicolor* [50,51]. It includes a bromotryptophan unit which is frequently found in sponges and lower marine invertebrates [52]. In addition to barettin and 8,9-dihydrobarettin, another analogue has been discovered in 2008: bromobenzisoxazolone barettin [53,54]. The authors pointed out that the three products are likely produced by a microbial symbiont in *G. barretti*, since sponges lack the shikimic acid pathway necessary to produce the tryptophan unit [53]. They were also identified in the species *G. macandrewii* [54].

The related compounds geobarrettin A–C have been isolated from *G. barretti*. Geobarrettins A and B are close analogues to barettin, whereas geobarrettin C bears a 6-bromoindole scaffold similar to BCC (Figure 2) [36]. A fourth derivative, geobarrettin D, has been isolated recently. The compound bears a bromo-indole moiety linked to a (purine-type) herbipoline unit. It has revealed a modest anti-inflammatory activity, reducing IL-10 secretion in matured dendritic cells. Biosynthetically, it would derive from the coupling of an oxidized bromo-tryptamine with a guanine unit [55]. Herbipoline (7,9-dimethylguanine betaine) is a natural product also isolated from *Geodia* sponges (e.g., the siliceous giant sponge *G. gigas* [56]) (Figure 2).

## 4. Chemical Synthesis of Barettin and Analogues

**Scheme 1:**
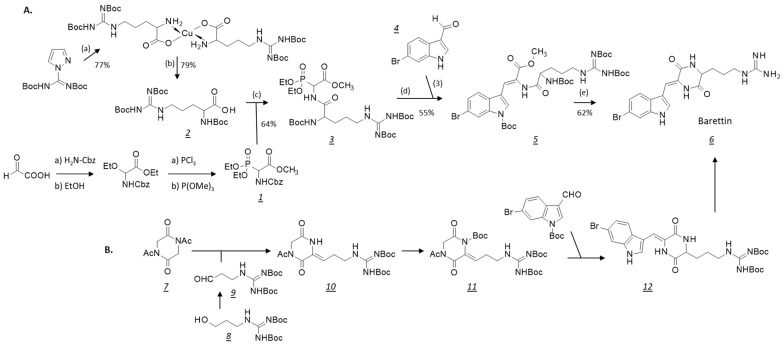
Synthesis of barettin. *Top* (**A**), the Horner–Wadsworth–Emmons-type process proposed by Johnson and coworkers in 2004 [57]. (a) Cu(Orn)_2_, CuCl_2_, DIEA, formamide/dioxane, rt, 5h. (b) EDTA, NaHCO_3_, Boc_2_O, H2O/acetone, rt, 12h. (c) (i) H_2_, Pd/C, EtOH, 4.5 h. (ii) [2], EDCI, HOBt, DIEA, CH_2_Cl_2_, rt 18 h. (d) *3*, DBU, CH_2_Cl_2_, −78° to rt 20 h. (e) (i) TFA, CH_2_Cl_2_, rt 12 h. (ii) 0.1 M HOAc/1-BuOH, NMM, reflux 6 h [57]. *Bottom* (**B**), the alternative route proposed by Kelley and coworkers in 2017. The stable intermediate *10* was prepared in three steps (Dess–Martin oxidation, aldol condensation, catalytic hydrogenation) from the N-Boc protected hydroxypropyl guanidine derivative *8* with a yield of 67%. After conversion of lactam *10* into imide *11* with Boc_2_O and DMAP, the product was condensed with the 6-bromoindole aldehyde derivative to obtain the desired aldol condensation product *12*. The final deprotection with TFA gave (±)-barettin *6* [59].

The total synthesis of the correct (guanidine-type) structure of barettin was accomplished in 2004 [57]. It consisted in the coupling of the indole-3-carboxaldehyde derivative *4* with a phosphonoglycinate derivative *3* in the presence of DBU (1,8-diaza-7-bicyclo[5.4.0]undecene) to obtain the condensation product *5*. The intermediate *3* was obtained upon hydrogenolysis of methyl 2-benzyloxycarbonylamino-2-(diethoxyphosphinyl)-acetate *1*, itself prepared from the condensation of an aldehyde with a N-acyl-2-(dialkyloxyphosphinyl)-glycine ester, via a Wittig–Horner-type P=O olefination reaction [58]. The Boc-protected arginine intermediate *2* was obtained from N-Boc 1-guanylpyrazole via a Cu complex precursor. The Boc-protecting groups of *5* were then removed to release barettin *6*, as depicted in Scheme 1A. This multistep procedure leads to the desired product but it is rather long and tedious, with an overall yield < 10%.

Kelley and coworkers have proposed a shorter route to the synthesis of barettin, starting from a protected hydroxypropyl guanidine derivative. The synthesis worked well (41% overall yield) and can be useful to prepare other mono-alkylidene diketopiperazines from bisacetoxyglycine anhydride [59] (Scheme 1B). The synthesis started with bisacetoxyglycine anhydride *7* coupled with β-guanidinyl aldehyde *9* (prepared from the protected hydroxypropylguanidine derivative *8*) to give the aldol condensation product *10*. The reduction of this aldol followed, with a second aldol condensation with 6-bromoindolecarboxaldehyde giving the bis-Boc protected intermediate *11*, which was finally deprotected with TFA (trifluoroacetic acid) to give barettin *6*. This 4-step sequence comprising two aldol condensations, a reduction, and lactam N-acylation works well, with a 41% overall yield starting from a protected hydroxypropyl guanidine derivative (the precursor to aldehyde *8*) (Scheme 1B). This general reaction sequence can be used to synthesize other mono-alkylidene diketopiperazines [59].

**Scheme 2:**
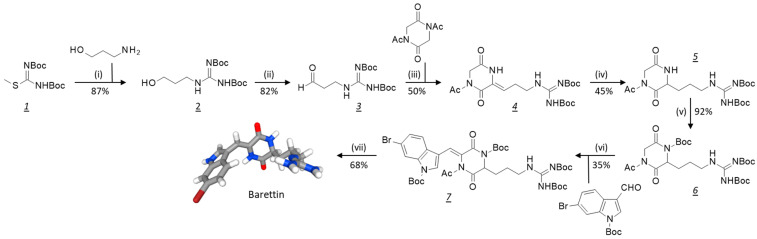
Synthesis of barettin according to [60]. (i) DIEA in CH_2_Cl_2_, (ii) DMP in pyridine/CH_2_Cl_2_, (iii) CsCO_3_ in DMF, (iv) H_2_/Pd-C, EtOAc, (v) Boc_2_O, DMAP/CH_3_CN, (vi) LiHMDS in THF, (vii) TFA/CH_2_Cl_2_.

A new synthetic route for barettin has been disclosed recently [60] (Scheme 2). The authors proposed an efficient and versatile process starting from bis-Boc protected S-methylthiourea *1* condensed with 3-aminopropanol to generate the guanidine-alcohol intermediate *2*. The primary alcohol is oxidized to an aldehyde *3* which is then coupled with glycine diacetyl anhydride to obtain the condensation product *4*. After hydrogenation of *4* and N-Boc-protection of the amide *5*, the intermediate *6* was coupled with 6-bromoindole-3-carboxaldehyde to give the N-Boc protected product *7*. After removal of the Boc groups, barettin was obtained with an overall yield of about 3%. The major advantage of this multistep procedure is its adaptability to prepare series of analogues incorporating various substituents on the diketopiperazine central motif (see below). The two synthetic processes can be exploited to prepare a diversity of barettin derivatives [59,60]. The first method starting from a protected hydroxypropyl guanidine and a 4-step sequence is particularly efficient and convenient to design a broad range of mono-alkylidene diketopiperazines [59].

**Scheme 3:**
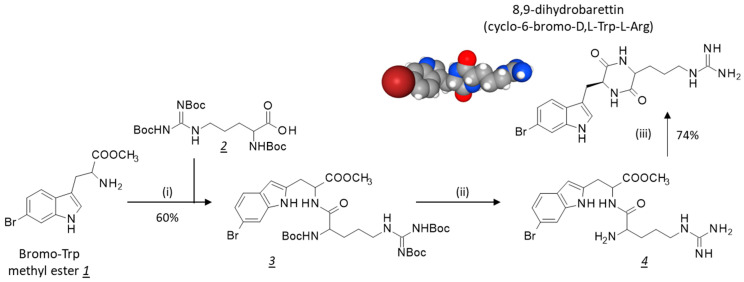
Synthesis of dihydrobarettin according to [57]. The synthesis started from 6-bromo-D,L-tryptophan to afford cyclo-6-bromo-D,L-Trp-L-Arg (or 8,9-dihydrobarettin) as a mixture of diastereomers. (i) coupling of *1* and *2* in the presence of EDCI, HOBt, DIEA in CH_2_Cl_2_, at rt for 18 h. (ii) Deprotection of *3* with TFA in CH_2_Cl_2_, at rt for 12 h. (iii) Cyclisation (DKP formation). 0.1 M HOAc/1-BuOH, N-methylmorpholine, at reflux for 6 h.

Dihydrobarettin was synthesized from 6-bromo-D,L-tryptophan, which is first methyl esterified (cpd *1* in Scheme 3) prior to coupling with the protected N-Boc arginine derivative *2* to obtain the corresponding dipeptide *3*. The removal of the protecting Boc groups with TFA and subsequent cyclisation afforded cyclo-6-bromo-D,L-Trp-L-Arg, which is 8,9-dihydrobarettin. The protected arginine derivative was prepared from N-protected amidino-pyrazole, via the Cu-complex intermediate, as depicted in Scheme 1A [57].

**Figure 4:**
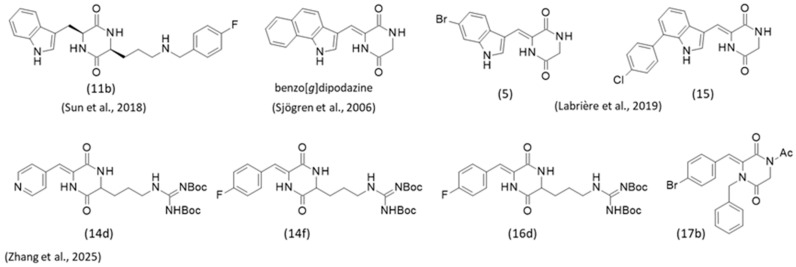
Structures of selected synthetic (dihydro)barettin derivatives which have revealed interesting biological activities. benzo[*g*]dipodazine is a potent antifouling agent [63]. Compound **11b** is a nematocidal agent [61]. Compounds **5** and **15** exhibit potent antioxidant activity [64]. **14d** and **14f** display marked anti-TMV activity, and **17b** binds to the TMV coat protein. **16d** showed an antifungal activity against *Phytophthora capsica* [60] (see text for details of bioactivities).

A series of synthetic dihydrobarettin derivatives lacking the bromine atom has been prepared and the compounds evaluated as anti-nematocidal agents. Over 70 piperazinedione derivatives were synthesized and tested against the root-knot nematode *Meloidogyne incognita* (a highly destructive parasite in chickpeas, eggplants, and other plants). A potent derivative (**11b** with a nematocidal activity of 75% at 2.4 μM) was identified, opening the route to the design of other active compounds in the dihydrobarettin series [61] (Figure 4).

In both barettin and dihydrobarettin series, synthetic analogues have been proposed over the last few years. Chronologically, the studies include the synthesis of the following:

(a) 5-bromobarettin, debromobarettin and various dipodazine derivatives, all evaluated as antifouling agents. The compound benzo[*g*]dipodazine, with a stronger activity than barettin, was discovered (EC_50_ = 0.034 and 0.9 µM, respectively) (Figure 4). This compound potently inhibited settlement of barnacle cyrpids larvae of *Balanus improvisus* (Crustacea), presumably via a specific binding to serotonergic 5-HT receptors [62]. Subsequently, about 30 derivatives of the lead compound benzo[*g*]dipodazine were synthesized incorporating alternative heterocyclic cores (e.g., hydantoin, rhodanine) but none of them revealed a high level of activity. Even the analogue benzo[*e*]dipodazine was found to be much less active than the parent benzo[*g*]dipodazine (EC_50_ = 0.03 and 1.7 µM, respectively). The natural 2,5-diketopiperazine ring is apparently not essential to the antifouling activity. The benzo[*g*]indole group is important, but not sufficient to confer activity [63].
(b) 2,5-diketopiperazines, such as the indole derivatives **5** and **15,** which exhibited an interesting level of activity in a HepG2 cell lipid peroxidation antioxidant activity (CLPAA) assay (Figure 4). These two compounds were more efficient than barettin (EC_50_ = 10, 10, 30 µM, respectively) [64]. Similar indole-diketopiperazines with antioxidant properties have been isolated from marine fungi (e.g., *Aspergillus* sp.) [65,66].
(c) Recently, a structural optimization of barettin has been proposed in the frame of a medchem study aimed at discovering new antiviral agents active against the tobacco mosaic virus (TMV) [60]. The replacement of the indole unit of barettin with a *p*-pyridine (**14d**) or a 4-fluorophenyl (**14f**) gave compounds with a potent anti-TMV activity, but only when the N-Boc protecting groups were maintained. The corresponding guanidine-type compounds were much less active. Two other analogues **16d** and **17b** also presented an interesting anti-TMV activity (IC_50_ = 220 and 214 µg/mL, respectively) (Figure 4). This later compound **17b** was shown to form stable complexes, with the TMV coat protein leading to protein disk aggregation and inhibition of viral assembly. This series of compounds were also evaluated as antifungal agents using *Physalospora piricola* and *Phytophthora capsica*. Here also, a few compounds of interest were identified, such as compound **16d** which was found to be more active than the reference chlorothalonil against *P. capsica* (IC_50_ = 7.54 and 10.77 µg/mL, respectively) [60].

These chemical studies provide a good basis to guide the design of other (dihydro)barettin derivatives, notably additional 2,5-diketopiperazines (DKPs) equipped with a guanidine or amidine group. It is interesting to note that structurally related compounds bearing a DKP motif connected with one or two amidine or guanidine groups have been identified as inhibitors of human β-tryptase, a serine protease with trypsin-like activity released from mast cell secretory granules [67]. 2,5-DKPs are versatile molecules which can be prepared by liquid- or solid-phase synthesis [68,69]. The DKP motif is largely represented in marine natural products [70,71] and the chemistry of 2,5-DKP has been well developed, enabling construction of combinatorial libraries [72]. These methods can be exploited to design new molecules inspired from barettin.

## 5. Bioactivity and Pharmacology of Barettin and Analogues

### 5.1. Antifouling Activity

In nature, barettin likely serves as a defense element against predators and as a sponge-protecting agent to avoid colonialization by other organisms [73]. The compound has been shown to inhibit settlement of the blue mussel *Mytilus edulis* a little more efficiently than its analogue 8,9-dihydrobarettin [45,74]. Barettin may also contribute to limiting the colonization of the sponge by planktonic foraminifera. The comparison of the colonization of 12 siliceous sponges revealed that *G. barretti* has the lowest density of foraminifera (3–14 ind./cm^3^) compared to other sponges [75]. Both barettin and 8,9-dihydrobarettin display antifouling activities [62] but their potency is modest compared to what can be achieved with related 2,5-diketopiperazines such as cyclo-L-Trp-L-Ala [76]. As mentioned above, the antifouling activity can be enhanced considerably upon modification of the core barettin structure. Benzo[*g*]dipodazine has been identified as a potent antifouling agent [62,63]. The mechanism at the origin of the antifouling activity of barettin is not totally clear at present. Apparently, it involves binding to and inhibition of serotonergic 5-HT (5-hydroxytryptamine) receptors in invertebrates by both barettin and 8,9-dihydrobarettin acting synergistically [46]. In mammalian species, barettin displays a high affinity to 5-HT_2A_, 5-HT_2C_, and 5-HT_4_ receptors, whereas 8,9-dihydrobarettin binds best to 5-HT_2C_ but has no affinity for 5-HT_2A_ and and 5-HT_4_ [74,77]. The difference between the two products has been attributed to the double bond in barettin which causes a more rigid steric orientation of the bromotryptophan residue, resulting in a better fit of the receptor-binding pocket of the serotonin receptors [77]. Barettin can form stable complexes with both 5-HT_2A/C_ receptors [78]. In the sponge, the two compounds barettin and 8,9-dihydrobarettin are excreted and function as serotonin receptor antagonists inhibiting the formation of biofilms (microfouling) and the limitation of macrofouling [79].

The capacity of barettin to bind 5-HT_2A/C_ receptors can be exploited to design antipain agents. A recent study revealed that an i.p. administration of barettin (32–178 mg/kg) induced a dose-dependent antinociceptive effect in a mouse model of chemotherapy-induced neuropathic pain. Its analgesic activity can be abolished with the concurrent administration of the 5-HT_2A/C_ antagonist ketanserin prior to barettin [78]. These observations strongly suggest that 5-HT_2A/C_ receptors represent primary targets for barettin, and certainly for its 8,9-dihydro analogue, but more work is needed to determine the main molecular target (5-HT_2A_ and/or 5-HT_2C_) and then to design orally-active 5-HT receptor antagonists. Barettin can be exploited to design novel antifouling agents but also novel modulators of neurotransmission. Efficient, affordable and eco-friendly anti-fouling agents and coatings remain needed [80].

**Figure 5:**
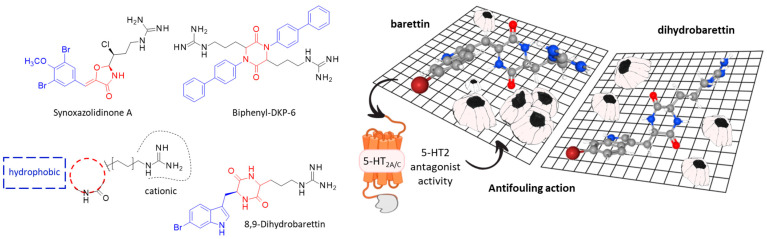
Schematic of antifouling activity of (dihydro)barettin acting as serotonin (5-HT2) antagonists. Structural analogy between barettins and DKP-6 derivative or natural product synoxazolidinone A. These products display potent antifouling activities [81,82].

At this point, it is worth refering to other antifouling agents with a barettin-like structure, such as the natural product synoxazolidinone A from the ascidian *Synoicum pulmonaria* [81] and the highly potent lead product biphenyl-DKP-6 [82]. These compounds present a common architecture with a central amide motif (DKP,oxazolidinone) connected with a bulky hydrophobic substituent on one side (dibromo-4-methoxybenzoyl, biphenyl, bromo-indole), and a guanidine (Y) cationic group on the other side (Figure 5). This amphiphilic organization has been exploited to design new antifouling agents inspired from nature [82,83,84,85].

### 5.2. Antioxidant and Anti-Inflammatory Activities

In addition to the above-mentioned activities against the TMV virus and phytopathogenic fungus *Physalospora piricola* (IC_50_ = 8.98 µg/mL) [60], barettin displays antioxidant and anti-inflammatory properties. The total antioxidant capacity (measured using a ferric reducing/antioxidant power (FRAP) assay) was found to be more important with barettin compared to debromobarettin. The difference was even more pronounced in a cell-based assay to measure lipid peroxidation. Barettin reduced lipid peroxidation by 55% at 30 µg/mL, whereas the synthetic analogue debromobarettin did not show any activity. Only the natural product was able to prevent lipid peroxidation within cellular membranes. In the same study, anti-inflammatory action was reported, through the capacity of barettin to inhibit secretion of interleukin-1β (IL-1β), and to a lower extent TNFα, from human THP-1 monocytes (at concentrations 50–100 µM) [86]. Later, the compound was shown to reduce, in a dose-dependent manner, the production of the anti-inflammatory cytokine IL-10 in lipopolysaccharide (LPS)-stimulated THP-1 macrophages. The effect was even more pronounced when THP-1 cells were co-stimulated with both LPS and IL-4 [87]. Barettin has been shown to reduce secretion of IL-10 and IL-12p40 in human dendritic cells (IC_50_ = 11.8 and 21.0 µM, respectively) but without an effect on the secretion of interferon IFN-γ or IL-17. The drug has an effect essentially on the Th1-type immune response [36].

The anti-inflammatory target of barettin is not known at present but the compound was found to inhibit kinases CAMK1α, SIK2, and RIPK2 (IC_50_ = 5.7, 6.1, 8.0 µM, respectively). The inhibition of CAMK1α (calcium/calmodulin-dependent protein kinase 1 α) could well occur at the origin of the observed blockade of IL-10 production [87]. This kinase inhibitory action provides novel options for the design of barettin analogues. However, one must admit that the potency is modest compared to existing nM-active inhibitors of RIPK2 and/or CAMK1 [88]. Other enzymes might be inhibited by barettin, notably proteases, as suggested in the frame of an in silico study aimed at identifying compounds active against COVID-19. Barettin was shown to bind to the two host proteases TMPRSS2 (trypsin-like transmembrane serine protease 2) and CTS-L (cathepsin-L). The coronavirus relies on these two proteins (and others) to enter into host cells. The blockade of these proteases might thus reduce the entry of the SARS-CoV-2 virus. In both cases, stable barettin-enzyme complexes were observed in silico, leading potentially to a dual inhibition of TMPRSS2 and CTS-L [89]. But no experimental validation has been provided thus far.

## 6. Conclusions and Perspectives

Marine siliceous sponges are widely distributed in nature and represent essential components of marine benthic communities. Among this group, demosponges of the genus *Geodia* (family Geodiidae) are well represented in the depths of the North Atlantic Ocean, notably around Scandinavian costs [90]. *Geodia* sponges play major roles in nutrient cycling, carbon turnover and the silicon cycle. Unfortunately, their vulnerable ecosystem is endangered due to excessive modification of the seafloor by various anthropogenic activities leading to increased CO_2_ emissions and ocean acidification. A decline of *G. barretti* has been observed in certain areas, for example in the Kosterhavet marine protected area in Sweden [91]. Measures have been taken to limit exposure of their habitats, notably via specific international conventions and agreements (e.g., NAFO, OSPAR, BBNJ) for the protection of the North-East Atlantic marine environment. Nevertheless, the populations of these demosponges tend to decrease. In particular, a recent study evidenced the major impact of bottom trawling on reducing the abundance and size of boreal *Geodia* spp. and *G. barretti* in particular [92]. Novel fishery regulations and protective measures (e.g., video surveillance combined with deep-learning technology) have been proposed to enhance protection of these cold-water species [93]. In the current context of global warming, it is important to take new measures to promote the protection of marine habitats, notably from epidemic diseases and human-driven activities.

*G. barretti* is known for its complex metabolism, with both aerobic and anaerobic processes, associated with its microbial symbionts. It is considered as a high-microbial-abundance (HMA) species, with a rich reservoir of bacterioplankton [94,95]. The microorganisms profit from the unique internal microstructure of the sponge skeleton to establish their niches [96]. The sponge creates a microenvironment favorable for the cell adhesion and proliferation of associated microorganisms. The prokaryotic community in the sponge contributes to the chemical diversity. The different diketopiperazine compounds found in *G. barretti* are likely implicated in bacterial communication and quorum sensing. The six analogous barettin-type products found in the sponge (barettin, 8,9-dihydrobarettin, 8,9-dihydro-8-hydroxybarrettin, bromobenzisoxalone barettin, and geobarrettins A-B) certainly play a central role to maintain the stability and security of the microbial environment in the sponge. Together with the other indole alkaloids, notably the four related compounds 6-bromo-8-hydroxyconicamin, 6-bromoconicamin, and geobarrettin C-D, barettin alkaloids contribute to the defense of the sponge against aggressors. Altogether, these 10 natural products serve as antifouling agents and regulators of a structured microbial environment in the sponge. They certainly contribute to maintaining the relationships between hosts and a proper equilibrium of the bacterial community in the sponge. The biogenic habitat formed between the sponge and its microbiome is adaptative as a function of depth. There is host-microbiome metabolic compensation, as demonstrated recently for the related species *G. hentscheli* [97].

The chemical environment of *G. barretti* warrants further research to identify new bioactive molecules, novel pharmacological properties and bio-applications. Thus far, research has been limited, in part due to the difficulty to collect the sponges from deep milieux. In recent years, the biotechnology of *Geodia* sponges has progressed significantly, in particular with the advent of in vitro marine cell cultures for biomass production. In vitro cultures of *G. barretti* have been developed with specific media, as well as the cryopreservation of cultured cells needed to inoculate new cultures [98,99,100]. The cultivation of sponge fragments has also been developed, enabling the production of sponge tissue in a fully controlled milieu [101]. These culture systems will facilitate the study of the sponge-microbiome symbioses, the scale-up production of known sponge-associated chemicals, and the discovery of new chemical regulators, even if the culture conditions can have an impact on the metabolite profile of the sponge [43]. The aquaculture of *G. barretti* sponges for the production of bioactive metabolites needs to be further developed.

At the chemical level, barettin is a useful natural model to guide the design of antifouling agents. Efficient, affordable and eco-friendly anti-fouling products and coatings remain needed [80]. Barettin is a source of inspiration for chemists, biologists and pharmacologists [102]. The chemistry of barettin has been well developed, leading to a reasonably good structural diversity in this DKP-type series. In contrast, the pharmacological knowledge about barettin targets and its mechanism of action is extremely limited. However, new ideas have been raised with the use of the application of in silico chemography-based tools such as the chemical global positioning system for natural products (ChemGPS-NP). Using this scoring tool, it has been suggested that barettin and related compounds can bind to vitronectin receptor alpha (integrin αVβ3), which is a key member of the integrin superfamily of adhesion molecules [103]. It remains a hypothesis at present but it makes sense because similar molecules with a 2,5-diketopiperazine scaffold have been shown to be integrin αVβ3 antagonists [104,105,106]. In addition, the marine natural product isoechinulin B, incorporating an indole-diketopiperazine core analog to that of barettin, has been shown recently to reduce expression of adhesion molecules and to inhibit cell adhesion between leukocytes and endothelial cells [107,108]. In the same vein, it is worth mentioning the marine indole alkaloid phidianidine A, which incorporates an 1,2,4-oxadiazole unit and a guanidine chain similar to that of barettin [109,110]. This compound functions as an antifouling agent and an antagonist of the chemokine receptor CXCR4 [111,112]. It also offers a source of inspiration for the design of other barettin analogues and the investigation of their pharmacological properties. Therefore, it can be recommended to extend studies on barettins in this direction. Hopefully, this review will encourage further studies on *G. barretti* and the discovery and pharmacological analyses of barettins and derivatives. This review offers the opportunity to take these natural products from the depths of knowledge and to bring them to the surface, with the hope of guiding future research in a creative manner.

## Data Availability

Data sharing is not applicable.

## Abbreviations

The following abbreviations are used in this manuscript:

BCC	Bromoconicamin
BHP	Bromohypaphorine
CLPAA	Cell lipid peroxidation antioxidant activity
CTS-L	Cathepsin-L
DKP	Diketopiperazine
FRAP	Ferric reducing/antioxidant power
HMA	High microbial abundance
TFA	Trifluoroacetic acid
TMV	Tobacco mosaic virus
TMPRSS2	Trypsin-like transmembrane serine protease 2
